# Socio-demographic determinants of Monkeypox virus preventive behavior: A cross-sectional study in Pakistan

**DOI:** 10.1371/journal.pone.0279952

**Published:** 2023-08-10

**Authors:** Hashaam Jamil, Muhammad Idrees, Kashmala Idrees, Waleed Tariq, Qudsia Sayyeda, Muhammad Sohaib Asghar, Muhammad Junaid Tahir, Samra Akram, Kaleem Ullah, Ali Ahmed, Aroop Mohanty, Bijaya Kumar Padhi, Ranjit Sah

**Affiliations:** 1 Lahore General Hospital, Lahore, Pakistan; 2 Multan Institute of Kidney Diseases, Multan, Pakistan; 3 Red Crescent of Tampa Bay, Tampa, FL, United States of America; 4 Mayo Clinic, Rochester, MN, United States of America; 5 Pakistan Kidney and Liver Institute and Research Center, Lahore, Pakistan; 6 Pir Abdul Qadir Shah Jeelani Institute of Medical Sciences, Gambat, Pakistan; 7 School of Pharmacy, Monash University, Subang Jaya, Selangor, Malaysia; 8 Department of Microbiology, All India Institute of Medical Sciences, Gorakhpur, India; 9 Department of Community Medicine and School of Public Health, Postgraduate Institute of Medical Education and Research, Chandigarh, India; 10 Tribhuvan University Teaching Hospital, Institute of Medicine, Kathmandu, Nepal; 11 Department of Microbiology, Dr. D. Y. Patil Medical College, Hospital and Research Centre, Dr. D. Y. Patil Vidyapeeth, Pune, Maharashtra, India; 12 Harvard Medical School, Boston, MA, United States of America; Virginia Commonwealth University, UNITED STATES

## Abstract

**Background:**

Monkeypox (mpox), re-emerging zoonotic infectious disease, is striking the world with serious public health concerns, especially in non-endemic countries. The public’s knowledge and attitude towards the monkeypox virus (MPXV) influence their adherence to preventive strategies. Therefore, we aimed to assess the public’s knowledge, attitudes, and perceptions (KAP) of MPXV in Pakistan.

**Methods:**

We collected data for this cross-sectional study from 1040 participants via online self-reported questionnaire from July 5^th^, 2022, to August 1^st^, 2022. The questionnaire consisted of a total of 29 items in four sections, assessing socio-demographics, knowledge, attitudes, and practices regarding MPXV. The data were analyzed using IBM SPSS V.25, and factors associated with MPXV knowledge, attitude, and practices were identified by using logistic regression analyses.

**Results:**

A total of 1040 participants were included. 61.4% were male, and 57.2% had graduation level education. Only 34.4% had good knowledge about MPXV, and 30% knew the effectiveness of the smallpox vaccine against MPXV. 41.7% had a positive attitude, 48.6% agreed that it is a fatal disease, and 44.6% were in favour of banning travel from endemic to non-endemic regions. 57.7% had good practices, and 69.9% would use protective measures if MPXV became an epidemic. Binary logistic regression analysis revealed that gender and education significantly impacted knowledge (p<0.05). While monthly income status had a significant impact on attitudes (p<0.05). The practices were positively correlated with gender and education (p<0.05).

**Conclusion:**

The majority of study participants had inadequate levels of knowledge, and attitudes regarding MPXV. To prevent the emergence and spread of MPXV in Pakistan, a comprehensive strategic framework for public health education must be established and implemented.

## Introduction

The monkeypox virus (MPXV), a member of the *POXVIRIDAE* family of the genus *Orthopoxvirus*, is the pathogen responsible for monkeypox (mpox) fever, a zoonotic infectious disease [1]. The first known outbreak of the virus in monkeys was discovered in 1958 at an animal research centre in Copenhagen, Denmark, giving rise to the term "monkeypox" [2]. MPXV was not recognized as a human virus until 1970 when the virus was isolated from a child in the Democratic Republic of the Congo (DRC) who had symptoms of suspected smallpox infection [3]. The potential modes of MPXV transmission are either animal-human or human-human. Animal-human transmission is associated with direct contact with the blood, body fluids, and cutaneous or mucosal wounds of infected animal hosts [4]. Close contact with respiratory secretions, infected patient objects or surroundings, an infected person’s skin lesions, and sexual contact with an infected person who has genital and groin lesions can cause human-to-human transmission [5]. However, in the recent multi-country MPXV outbreak, the majority of MPXV cases have primarily been reported among men who have had sex with men (MSM) [6–8].

Human mpox has clinical features that are strikingly similar to those of smallpox, but less severe [9, 10]. After an incubation period of 10 to 14 days, the majority of patients experience prodromic symptoms, including fever, malaise, and enlarged lymph nodes. About 90% of patients infected with MPXV develop lymphadenopathy [11, 12]. After an initial prodromal period, a centrifugally distributed maculopapular rash developed. Lesions develop from macules to papules, vesicles, pustules, and eventually crust over in two to four weeks [13]. Treatment of MPXV is mainly symptomatic. The smallpox vaccination is 85% effective in protecting against mpox [14]. However, Modified Vaccinia Ankara-Bavarian Nordic (MVA-BN), an authorized vaccine, and the antiviral medications tecovirimat and brincidofovir are not easily available [15, 16].

In 2003, the United States reported the first MPXV outbreak in humans outside of Africa, with 70 cases, which was associated with the importation of exotic pets from Ghana [17]. Since then, the incidence in endemic areas has substantially increased, and sporadic, travel-related incidents are on exponential rise in non-endemic nations [13, 18]. In September 2017, Nigeria reported a major outbreak of human MPXV, with about 228 suspected and 60 confirmed cases caused by the West African clade [19]. In the ongoing outbreak, the UK Health Security Agency reported the first mpox case on May 07, 2022. Since that time, the World Health Organization (WHO) has consistently recorded reports of mpox cases [20, 21]. Mpox was declared a public health emergency of international concern by the WHO on July 23, 2022, due to an upsurge in cases [22]. From January 1^st^ through November 17^th^, 2022, 80221 confirmed cases of MPXV via PCR and 52 mortalities have been reported to the WHO from 110 countries [23]. Although MPXV has been reported in other southeast Asian countries (India, Thailand, and Indonesia) [24, 25], so far, Pakistan has not reported any cases of MPXV. Although, there were reports on two rarely occurring cases of the zoonotic mpox infection [26], but the reporting of mpox cases in Pakistan was false news, which was refuted by the National Institute of Health on May 24, 2022 [27].

According to the WHO, a lack of awareness about the virus is one of the obstacles in preventing the re-emergence of MPXV [28]. Adherence to prevention and control initiatives is important in restricting the dissemination of the virus, which is largely influenced by the community’ cumulative knowledge, attitudes, and practices (KAP) [29, 30]. A comprehensive strategic framework must be established and implemented to prevent the emergence and spread of MPXV in Pakistan. Our findings emphasize the need to investigate the KAP towards MPXV among the general population of Pakistan. The study’s conclusions could assist public health officials in strengthening policies, increasing public awareness of the MPXV outbreak, and organizing future public health initiatives.

## Methods

### Study design, setting and data collection

An analytical cross-sectional study was applied to assemble data from Pakistan’s general population using a non-probability convenience sampling technique. Using shared Google forms links across WhatsApp, Instagram, Facebook, and Twitter, Pakistan’s most widely used and accessible social media platforms, the research team developed a questionnaire with informed consent. Between July 5^th^, 2022, to August 1^st^, 2022, data were collected across all of Pakistan’s provinces (i.e., Sindh, Punjab, Baluchistan, and Khyber Pakhtunkhwa). Individuals below the age of 18, non-Pakistanis, and those who were unable to understand the questionnaire language were excluded from the study.

### Sample size

Pakistan’s population, according to the most recent census, is 207.7 million [31]. Using a Raosoft sample size calculator online, minimum sample size of 752 was calculated using a 90% confidence interval, 50% of the population, a 3% margin of error, and a total population of 207.774 million. We ultimately obtained 1040 responses to include in the survey, which will help us get more accurate and reliable results.

### Questionnaire development

The self-reported questionnaire was developed using an extensive literature review [16, 32, 33] and information was sorted out from the WHO and Centers for Disease Control and Prevention’s official websites (CDC) [34, 35]. This survey form was reviewed by a team comprising senior community medicine specialists, public health experts, and infectious disease researchers to improve some details for the pseudo-validity, relevance, completeness, and clarity of individual sections. After a detailed discussion and audit, the authors finalized the survey. Then, 80 people participated in pilot research to examine the reliability of the system. Cronbach’s alpha was reported to be 0.76, and the results of the pilot study were not included in the final analysis.

The beginning of questionnaire was introduced with a brief overview of the nature and purpose of the study, informed consent, and then segregated into four divisions to assess the socio-demographics of the participants, knowledge, attitudes, and practices. Six questions regarding age, gender, marital status, residence, education, and monthly income were included in the demographic section.

The second section evaluated the knowledge of participants of mpox by asking 10 questions. Each score was graded as “1” (correct) and “0” (wrong) with the scores ranging from 0 to 10. The study participants who scored 7 or more were classified as having good knowledge.

The third section assessed attitudes. Seven questions were tested by a 5–point Likert scale as follows: 1 (“strongly disagree”), 2 (“disagree”), 3 (“neutral”), 4 (“agree”), and 5 (“strongly agree”). The overall score for the attitude section ranged from 7 to 35, with an overall higher score depicting a more positive attitude. A score higher than or equal to 26 was described as a positive attitude.

The last section assessed the practices. Six questions were tested with a 5–point Likert scale as follows: 1 (“strongly disagree”), 2 (“disagree”), 3 (“neutral”), 4 (“agree”), and 5 (“strongly agree”). The practice section score ranged from 6 to 30, with an overall higher score depicting a more positive practice. A score more than or equal to 22 was considered a positive practice.

### Ethical considerations

The anonymity and confidentiality of participants were ensured during the survey. The research did not include names or other personal information. Before beginning the questionnaire, informed consent was taken from the sampled study participants. During the process of survey, participants can deny and withdraw at any time before submission. The ethical review committee of the Pir Abdul Qadir Shah Jeelani Institute of Medical Sciences in Gambat, Pakistan, approved the study protocol (Ref. No: IRB/22/13).

### Statistical analysis

The statistical package for social sciences (SPSS) version 25 was used for data analysis (IBM). While categorical variables were expressed as frequencies and percentages, numerical variables were quantified as means and standard deviations. Based on the variables and the data type, inferential statistics were used. Univariate logistic regression analysis carried out a comparison of knowledge, attitude, and practices with socio-demographic variables. The statistical results were demonstrated as odds ratio (OR) with a 95% confidence interval (CI) and p-value. In multivariable analysis, all with p-value <0.25 were included, and an adjusted odds ratio (aOR) was reported with independent association. A p-value of <0.05 was considered statistically significant.

## Results

### Socio-demographic characteristics

A total of 1040 participants were involved in the final analysis. Of the total, 61.4% (n = 639) were male, 61.8% (n = 643) fell in the age group 21–30 years, 79.5% (n = 827) were single. 79.5% (n = 789) of the respondents were residents of urban areas, and 57.2% (n = 595) achieved graduation level education (Table 1).

**Table 1:** Study sample socio-demographic characteristics (N = 1040).

Variables	Categories	Frequency	Percentage
Gender			
	Male	639	61.4
	Female	401	38.6
Age (years)			
	<20	234	22.5
	21–30	643	61.8
	31–40	124	11.9
	>40	39	3.8
Marital Status			
	Married	213	20.5
	Unmarried	827	79.5
Residence			
	Urban	789	75.9
	Rural	251	24.1
Education			
	Primary up to Matriculation	38	3.7
	Intermediate	197	18.9
	Graduation	595	57.2
	Post-graduation	210	20.2
Monthly incomein PKR (USD)			
	<50000 (225)	644	61.9
	50000–100,000 (225–450)	252	24.2
	>100,000 (450)	144	13.8

PKR: Pakistani Rupee.

### General knowledge, attitudes, and practices about Mpox

This study determined that 34.4% (n = 358) of the participants had good knowledge, while 65.6% (n = 682) had poor knowledge about MPXV. 47.7% (n = 496) respondents correctly answered that MPXV was first isolated in Congo, and 87% (n = 905) correctly identified the causative agent of mpox is virus. 65% (n = 683) knew about preventions and 64% (n = 668) were familiar with the symptoms. Only 30% (n = 312) of participants were familiar with the fact that the Smallpox vaccine is also effective against MPXV (Table 2).

**Table 2:** Knowledge of general population regarding Mpox virus (N = 1040).

Variables	Categories	Frequency	Percentage
Mpox disease was first isolated in?			
	Incorrect	544	52.3
	Correct	496	47.7
The causative factor of mpox disease is?			
	Incorrect	135	13.0
	Correct	905	87.0
Transmission of mpox disease caused by?			
	Incorrect	491	47.2
	Correct	549	52.8
Human-to-human transmission caused by?			
	Incorrect	554	53.3
	Correct	486	46.7
The incubation period (interval from infection to onset of symptoms)is?			
	Incorrect	424	40.8
	Correct	616	59.2
What are ways to prevent the spread of mpox?			
	Incorrect	357	34.3
	Correct	683	65.7
Common symptoms of mpox disease are?		-	
	Incorrect	372	35.8
	Correct	668	64.2
The case fatality ratio of mpox has historically ranged from?			
	Incorrect	471	45.3
	Correct	569	54.7
Preferred laboratory test for mpox diagnosis?			
	Incorrect	543	52.2
	Correct	497	47.8
Is there any vaccine available for mpox?			
	Incorrect	728	70.0
	Correct	312	30.0

Knowledge section was assessed by giving a score of 1 to correct answer and 0 to wrong answer. A score of greater than equal to 7 was regarded as good knowledge (N = 358{34.4%}).

Regarding attitudes, 41.7% (n = 434) of the participants had positive attitudes. 48.6% (n = 505) agreed that MPXV is a fatal disease, and 38.3% (n = 398) agreed that MPXV can cause a pandemic like the Coronavirus disease 2019 (COVID-19). 44.6% (n = 464) believed that traveling from endemic areas (such as Africa, Nigeria, etc.) to non-endemic areas should be banned (Table 3).

**Table 3:** Attitude of general population regarding Mpox virus (N = 1040).

Variables	SD	D	N	A	SA	Average
Do you believe that mpox is a fatal disease?	16 (1.5)	105 (10.1)	179 (17.2)	505 (48.6)	235 (22.6)	3.81
Do you believe that you are at risk of getting the disease?	68 (6.5)	190 (18.3)	333 (32.0)	351 (33.8)	98 (9.4)	3.21
Do you believe that mpox can cause a pandemic like COVID-19?	49 (4.7)	246 (23.7)	218 (21.0)	398 (38.3)	129 (12.4)	3.30
Do you believe that there should be a lockdown in areas reporting cases of mpox disease?	44 (4.2)	175 (16.8)	211 (20.3)	422 (40.6)	188 (18.1)	3.51
Do you believe that travelling from endemic areas (such as Africa, Nigeria,etc.) to non-endemic areas should be banned?	24 (2.3)	136 (13.1)	189 (18.2)	464 (44.6)	227 (21.8)	3.71
Do you believe that there is an increased risk of people getting the disease during Eid-ul-Adha?	30 (2.9)	136 (13.1)	214 (20.6)	478 (46.0)	182 (17.5)	3.62
Do you believe that small pox vaccine is effective against mpox?	33 (3.2)	128 (12.3)	377 (36.3)	400 (38.5)	102 (9.8)	3.39
**Overall attitude**						
	Negative (7–25)			606 (58.3%)		
	Positive (26–35)			434 (41.7%)		

Data presented as frequency (percentage), and average scores (mean) per total participants. SD = strongly disagree, D = Disagree, N = Neutral, A = Agree, SA = strongly agree. Attitude section was assessed by giving a score of 1 to strongly disagree and 5 to strongly agree. A score of greater than or equal to 26 was regarded as positive attitude.

Assessment of practices showed that the majority of participants, 57.7% (n = 600), had good practices. About half (48.0%) of respondents agreed that infected patients should be isolated from others. 38.9% (n = 405) strongly agreed that there is a need for more awareness of MPXV in the general public. When asked whether the government of Pakistan is taking adequate steps to tackle MPXV, 27.0% (n = 281) were neutral to making the decision (Table 4).

**Table 4:** Practices of general population regarding Mpox virus (N = 1040).

Variables	SD	D	N	A	SA	Average
Will you take precautionary measures if mpox disease becomes an epidemic in your area?	135 (13.0)	80 (7.7)	98 (9.4)	383 (36.8)	344 (33.1)	3.69
Quarantine is one way to prevent its spread at population levels just like COVID-19?	94 (9.0)	123 (11.8)	239 (23.0)	456 (43.8)	128 (12.3)	3.38
Infected patients should be isolate from others?	85 (8.2)	56 (5.4)	113 (10.9)	499 (48.0)	287 (27.6)	3.81
Avoiding crowds, using facemask and Washing hands frequently will prevent its spread?	84 (8.1)	74 (7.1)	178 (17.1)	508 (48.8)	196 (18.8)	3.63
Do you believe that there is a need of more awareness in general public about mpox?	90 (8.7)	59 (5.7)	99 (9.5)	387 (37.2)	405 (38.9)	3.92
Do you think government of Pakistan is taking sufficient steps to tackle with mpox?	174 (16.7)	332 (31.9)	281 (27.0)	195 (18.8)	58 (5.6)	2.64
**Overall practice**						
	Poor (6–21)			440 (42.3%)		
	Good (22–30)			600 (57.7%)		

Data presented as frequency (percentage), and average scores (mean) per total participants. SD = strongly disagree, D = Disagree, N = Neutral, A = Agree, SA = strongly agree. Practice section was assessed by giving a score of 1 to strongly disagree and 5 to strongly agree. A score of greater than or equal to 22 was regarded as good practice.

### Factors of good knowledge, attitudes, and practices

The regression analysis revealed that men had a higher odds ratio than women [Adjusted odds ratio (aOR) = 1.324; 95% Confidence interval (CI) = 1.001–1.752, p = 0.049]. Participants with an education level from primary up to matriculation (Class-10) were 0.22 times less likely to have good knowledge than those with intermediate education (Class-12) (aOR = 0.220; 95% CI = 0.074–0.653, p = 0.006) (Table 5).

**Table 5:** Multivariate logistic regression for good knowledge of study participants.

Variables	Knowledge		OR	95% CI	P-Value	aOR	95% CI	P-Value
	Poor knowledge	Good knowledge						
**Gender**								
Male	403 (63.1)	236 (36.9)	1.339	1.026–1.748	0.032*	1.324	1.001–1.752	0.049*
Female	279 (69.6)	122 (30.4)	Ref	-	-	Ref	-	-
**Age**								
<20	154 (65.8)	80 (34.2)	1.732	0.784–3.824	0.174	1.769	0.765–4.090	0.182
21–30	423 (65.8)	220 (34.2)	1.734	0.809–3.716	0.157	1.667	0.751–3.699	0.209
31–40	75 (60.5)	49 (39.5)	2.178	0.952–4.981	0.065	1.853	0.793–4.330	0.154
>40	30 (76.9)	9 (23.1)	Ref	-	-	Ref	-	-
**Marital Status**								
Married	138 (64.8)	75 (35.2)	1.045	0.762–1.433	0.786	-	-	-
Unmarried	544 (65.8)	283 (34.2)	Ref	-	-	-	-	-
**Residence**								
Rural	154 (61.6)	97 (27.1)	1.274	0.949–1.710	0.106	1.214	0.892–1.654	0.218
Urban	528 (66.9)	261 (33.1)	Ref	-	-	Ref	-	-
**Education**								
Primary up to Matriculation	34 (89.5)	4 (10.5)	0.213	0.073–0.626	0.005*	0.220	0.074–0.653	0.006*
Intermediate	127 (64.5)	70 (35.5)	Ref	-	-	Ref	-	-
Graduation	396 (66.6)	199 (33.4)	0.912	0.650–1.278	0.592	0.923	0.637–1.337	0.671
Post-graduation	125 (59.5)	85 (40.5)	1.234	0.826–1.843	0.305	1.287	0.801–2.067	0.296
**Monthly income in PKR (USD)**								
<50000 (225)	436 (67.7)	208 (32.3)	0.897	0.613–1.312	0.575	-	-	-
50000–100,000 (225–450)	152 (60.3)	100 (39.7)	1.237	0.808–1.894	0.328	-	-	-
>100,000 (450)	94 (65.3)	50 (34.7)	Ref	-	-	-	-	-

OR = Odds Ratio, CI = Confidence interval.

A p value of less than 0.05 considered statistically significant.

With respect to the different variables affecting attitudes regarding MPXV, and based on statistical values obtained by multivariate logistic regression, participants with an income of 50,000–100,000 Pakistani Rupees (PKR) {225–450 USD} had lower odds of positive attitudes compared to participants who had an income of more than 100,000 PKR {450 USD}, (aOR = 0.593; 95% CI = 0.384–0.917, p = 0.019), while those aged 31–40 years had a more positive attitude (aOR = 2.454; 95% CI = 1.099–5.483, p = 0.029) (Table 6). With regards to good practices, males had lower odds of good practices than females (aOR = 0.741; 95% CI = 0.572–0.959, p = 0.023).

**Table 6:** Multivariate logistic regression analysis for positive attitude of study participants.

Variables	Attitude		OR	95% CI	P-Value	aOR	95% CI	P-Value
	Negative	Positive						
**Gender**								
Male	375 (58.7)	264 (41.3)	0.957	0.743–1.232	0.731	-	-	-
Female	231 (57.6)	170 (42.4)	Ref	-	-	-	-	-
**Age**								
<20	142 (60.7)	92 (39.3)	1.649	0.783–3.474	0.188	1.390	0.634–3.049	0.411
21–30	369 (57.4)	274 (42.6)	1.890	0.925–3.863	0.081	1.900	0.895–4.035	0.095
31–40	67 (54.0)	57 (46.0)	2.166	0.991–4.732	0.053	2.454	1.099–5.483	0.029*
>40	28 (71.8)	11 (28.2)	Ref	-	-	Ref	-	-
**Marital Status**								
Married	124 (58.2)	89 (41.8)	1.003	0.739–1.361	0.986	-	-	-
Unmarried	482 (58.3)	345 (41.7)	Ref	-	-	-	-	-
**Residence**								
Rural	134 (53.4)	117 (46.6)	1.300	0.977–1.730	0.072	1.266	0.945–1.697	0.114
Urban	472 (59.8)	317 (40.2)	Ref	-	-	Ref	-	-
**Education**								
Primary up to Matriculation	20 (52.6)	18 (47.4)	1.048	0.523–2.102	0.894	0.987	0.478–2.037	0.972
Intermediate	106 (53.8)	91 (46.2)	Ref	-	-	Ref	-	-
Graduation	355 (59.7)	240 (40.3)	0.787	0.569–1.089	0.149	0.710	0.495–1.018	0.062
Post-graduation	125 (59.5)	85 (40.5)	0.792	0.535–1.173	0.245	0.787	0.489–1.265	0.322
**Monthly income in PKR** (USD)								
<50000 (225)	348 (54.0)	296 (46.0)	1.191	0.826–1.717	0.349	1.145	0.777–1.686	0.493
50000–100,000 (225–450)	174 (69.0)	78 (31.0)	0.628	0.410–0.960	0.032*	0.593	0.384–0.917	0.019*
>100,000 (450)	84 (58.3)	60 (41.7)	Ref	-	-	Ref	-	-

OR = Odds Ratio, aOR = Adjusted Odds Ratio, CI = Confidence interval.

A p value of less than 0.05 considered statistically significant.

## Discussion

To the best of our knowledge, this is the first study carried out in Pakistan to assess the KAP towards MPXV among the general population. Based on our results, most participants had poor knowledge and attitudes but positive practices toward MPXV. Gender and education had a significant impact on knowledge and practices. While monthly income status had a significant impact on attitudes.

Our study findings indicate that most study participants (65.6%) had poor knowledge about MPXV. This inference is consistent with other recent research that revealed that people generally in Saudi Arabia, Bangladesh, and the Kurdistan region of Iraq had little knowledge of MPXV [16, 36, 37]. As MPXV is endemic in tropical rainforest regions, Pakistan lies in a temperate climate zone, and there has been no previous exposure to the disease in the country, rendering the disease unknown among the general population [12, 38]. The majority were unaware of the origin of MPXV in the DRC. People visiting the endemic regions should be aware of the locally endemic diseases, which will help in the adoption of different preventive and treatment strategies, such as MPXV and COVID-19, which are endemic in the DRC and China, respectively [39, 40]. Thus, it is necessary to initiate awareness campaigns in Pakistan, especially for professional travelers, the business community, and tourists to the endemic region, to prevent the spread of disease to non-endemic areas.

However, participants in this study had good knowledge about the causative factor of mpox fever (87.0%), prevention of the disease (65.0%), and common symptoms (64%) (Table 7). Except for a few variations, smallpox, and mpox viruses belong to the same family, and many similarities are found in terms of disease presentation, spread, prevention, control, and management plans [41–43]. This might be one reason for being known common symptomatology of MPXV infection and preventive measures. But only 30% knew that the smallpox vaccine is also effective against MPXV. Vaccine against smallpox is considered beneficial for the prevention and control of MPXV [44, 45]. Some analyses also depicted that the MPXV outbreak was due to a break in vaccination against smallpox [46, 47].

**Table 7:** Multivariate logistic regression analysis for good practices of study participants.

**Variables: Knowledge about Mpox virus**	Value: **N(%)**
**Mpox disease was first isolated in?**	
Nigeria	269(25.9)
**Congo**	**496(47.7)**
China	224(21.5)
Japan	51(4.9)
**The causative factor of mpox disease is?**	
Bacteria	83(8.0)
**Virus**	**905(87.0)**
Fungus	33(3.2)
Parasite	19(1.8)
**Transmission of mpox disease occur by?**	
Human-to-human	200(19.2)
Animal-to-human	226(21.7)
Contact with contaminated material	65(6.3)
**All**	**549(52.8)**
**Human-to-human transmission occurs by?**	
Respiratory secretions of an infected person or animal	220(21.2)
Skin lesions of an infected person	227(21.8)
Contact with contaminated objects	78(7.5)
Feces or urine of infected person or animal	29(2.8)
**All**	**486(46.7)**
**The incubation period (interval from infection to onset of symptoms)?**	
1–5 days	237(22.8)
**6-13days**	**616(59.2)**
14-30days	172(16.5)
1–2 months	15(1.4)
**What are ways to prevent the spread of mpox?**	
Avoid contact with animals that could harbor the virus	109(10.5)
Practice good hand hygiene after contact with infected animals or humans.	161(15.5)
Isolate infected patients from others	87(8.4)
**All**	**683(65.7)**
**Common symptoms of mpox disease?**	
Loose motions, abdominal pain, and vomiting	159(15.3)
**Fever, headache, swelling of the lymph nodes, and muscle aches**	**668(64.2)**
Fever, cough and shortness of breath	120(11.5)
Fever, sore throat and cough	93(8.9)
**The case fatality ratio of mpox has historically ranged from?**	
**0–11%**	**569(54.7)**
15–30%	310(29.8)
40–50%	108(10.4)
More than 70%	53(5.1)
**Preferred laboratory test for mpox diagnosis?**	
Serology	165(15.9)
Complete blood test (CBC)	206(19.8)
Diagnosis is clinical, Need no laboratory confirmation	172(16.5)
**PCR (Polymerase chain reaction)**	**497(47.8)**
**Is there any vaccine available for mpox?**	
**Smallpox vaccine is also effective against mpox**	**312(13.1)**
Vaccine against mpox is available.	273(26.3)
No vaccine is available	455(43.8)

OR = Odds Ratio, aOR = Adjusted Odds Ratio, CI = Confidence interval.

A p value of less than 0.05 considered statistically significant.

Our study shows that most participants (58.3%) had a negative attitude toward MPXV, which is consistent with a study conducted on the general public’s attitude toward mpox in the United States [48]. Good knowledge is not beneficial without applying that knowledge in practical life. Individual knowledge can be practically utilized to adopt a positive attitude by engaging people in awareness activities. Studies in Iran and Indonesia about avian influenza postulated similar aspects of the correlation of knowledge with attitudes [49, 50]. Most respondents believed that MPXV is a fatal disease that can cause a pandemic like COVID-19. The COVID-19 pandemic hit many people hrad by evolving a lot of complications for them, and based on this, it might be possible they are considering MPXV to be fatal and a pandemic in the future [51, 52]. Some studies depict the role of the fatality of disease over one’s psychology as being the case with the Ebola virus disease [53, 54]. However, little information is available regarding the connection between MPXV and psychological issues [55].

In our study, most respondents had good practices compared to knowledge and attitudes. The findings are consistent with the studies conducted on general physicians showing good practices [32, 56]. In our study, most participants believed that infected patients should be isolated from others and that preventive measures such as face masks, hand washing, and quarantine would prevent the spread of MPXV. This might be due to the recent pandemic of COVID-19, during which taking precautionary measures was the sole mode of protection all over the world before the vaccine’s introduction [57]. The other reason for good practices could be recent alerts from WHO and CDC.

In agreement with the previous study [36], our study reported significantly good knowledge of MPXV among male participants. This may be connected to how MPXV is transmitted because the majority of cases in the most recent outbreak have mostly been reported among men who have had sex with men (MSM) [58, 59]. According to a recent pooled meta-analysis, sexual contact is involved in more than 91% of cases [6]. A study reported that all (100%) of the 54 MPX cases that presented at one health center in the UK were MSM [60]. Study findings affirm that respondents with higher education levels had better knowledge than those participants with qualifications of primary to matriculation (Class-10). Other studies with similar findings also revealed that a greater level of education was related to having good knowledge [61, 62]. Education has a key role in promoting healthy behaviors and gaining control over epidemics, by improving knowledge levels about any health-related case. Probably, this is because of more exposure to media, internet, and newspapers that provide easy access to knowledge and a higher maturity level. However, the association of age, residence, and monthly income with knowledge did not withstand the regression analysis in the current study.

Nevertheless, our results showed that the population with a low income had a more positive attitude. This is not consistent with many studies that revealed having a higher socioeconomic position corresponds with having a more positive attitude [63, 64]. People with higher socioeconomic status have additional opportunities for higher education, residing in urban areas, and exposure to mass media. The study findings demonstrate that females were shown to have more good practices compared to males. Similar findings were observed in other studies showing that females take more preventive measures than males [65]. Women claimed to be more responsible for their health, potentially related to risk perception and the gender bias that women are socialized to be more concerned about health issues than men.

Mpox is an emerging virulent disease poses a serious threat to worldwide public health [42]. Given the substantial risks posed by MPXV and the lack of mpox vaccine, prevention strategies play a crucial role in minimizing infection rates and halting the disease’s spread. It has been acknowledged that increasing awareness through campaigns and seminars, as well as embracing preventive strategies can halt infectious diseases like COVID-19, influenza, and dengue from becoming epidemics or pandemics [66–68]. Studies conducted to assess knowledge and attitudes about disease prevention and improve public health interventions are futile without public interest and collaboration [32, 69]. This highlights how crucial it is for the general public to follow preventative and control measures based on their knowledge, attitudes, and practice (KAP).

## Study strengths and limitations

While interpreting the study’s findings, some limitations should be considered. First, there was a possibility of selection bias because the convenience sampling method was used. Second, the questionnaire was distributed through an online system using different social media platforms. Consequently, there is a possibility of bias as underprivileged populations would not have been able to participate, and results cannot be extended to the entire community. Third, this study is based on self-reported data, which might lead to internet surfing to acquire information for a difficult and technical question in the survey, resulting in a biased response to some questions. According to what they believe to be expected, participants may have responded positively to attitude and practice questions. Fourth, a recall bias during the survey cannot be ignored. Finally, results stratified by marital status and residence should be interpreted with caution, since the majority of the participants were unmarried and urban. However, the current study was strengthened by the large sample size. To the researchers’ knowledge, this is the first study analyzing KAP toward MPXV, in the Pakistan’s public. Therefore, the results of this study may help health authorities to implement effective policies to combat the emergence of MPXV in Pakistan.

## Conclusion

In summary, the present study demonstrated inadequate knowledge and attitudes regarding MPXV among the Pakistani population. Our results showed that good knowledge could result in good attitudes and practices, which are crucial to minimizing the expanding impacts of the disease. The current outbreak of MPXV in non-endemic countries warrants strict epidemiological surveillance to restrict the further dissemination of the outbreak in other non-endemic countries. International organizations, national health authorities, national organizations, health care workers, and the media sector must be actively involved in the implementation of appropriate interventions for the awareness and prevention of MPXV.

## Supporting information

S1 File (SAV)

## References

[1] Kaler J, Hussain A, Flores G, Kheiri S, Desrosiers D. Monkeypox: A Comprehensive Review of Transmission, Pathogenesis, and Manifestation. Cureus. 2022;14(7):e26531. doi: 10.7759/cureus.26531 35928395 PMC9345383

[2] de Menezes Filho ACP, Ventura MVA, Batista-Ventura HRF, de Souza Castro CF, Teixeira MB, Soares FAL. Monkeypox cases in Brazil, a possible pandemic? Brazilian Journal of Science. 2022;1(10):1–4.

[3] Moore M, Zahra F. Monkeypox. StatPearls [Internet]: StatPearls Publishing; 2021.

[4] Alakunle E, Moens U, Nchinda G, Okeke MI. Monkeypox Virus in Nigeria: Infection Biology, Epidemiology, and Evolution. Viruses. 2020;12(11). doi: 10.3390/v12111257 33167496 PMC7694534

[5] Petersen E, Kantele A, Koopmans M, Asogun D, Yinka-Ogunleye A, Ihekweazu C, et al. Human Monkeypox: Epidemiologic and Clinical Characteristics, Diagnosis, and Prevention. Infectious disease clinics of North America. 2019;33(4):1027–43. doi: 10.1016/j.idc.2019.03.001 30981594 PMC9533922

[6] Sah R, Abdelaal A, Reda A, Katamesh BE, Manirambona E, Abdelmonem H, et al. Monkeypox and Its Possible Sexual Transmission: Where Are We Now with Its Evidence? Pathogens (Basel, Switzerland). 2022;11(8). 10.3390/pathogens11080924 PMC9414346 36015044

[7] Rodriguez-Morales AJ, Lopardo G. Monkeypox: Another Sexually Transmitted Infection? Pathogens (Basel, Switzerland). 2022;11(7). 10.3390/pathogens11070713 PMC9318491 35889960

[8] Iñigo Martínez J, Gil Montalbán E, Jiménez Bueno S, Martín Martínez F, Nieto Juliá A, Sánchez Díaz J, et al. Monkeypox outbreak predominantly affecting men who have sex with men, Madrid, Spain, 26 April to 16 June 2022. Euro surveillance: bulletin Europeen sur les maladies transmissibles = European communicable disease bulletin. 2022;27(27). doi: 10.2807/1560-7917.ES.2022.27.27.2200471 35801519 PMC9264731

[9] Taseen S, Nasir F, Abbas M, Altaf M, Asghar MS, Tahir MJ. Post-pandemic world at the mercy of monkeypox virus outbreak: Time to worry or not? J Med Virol. 2022. 10.1002/jmv.27948 35730397

[10] Nalca A, Rimoin AW, Bavari S, Whitehouse CA. Reemergence of monkeypox: prevalence, diagnostics, and countermeasures. Clinical infectious diseases: an official publication of the Infectious Diseases Society of America. 2005;41(12):1765–71. 10.1086/498155 16288402

[11] Quarleri J, Delpino MV, Galvan V. Monkeypox: considerations for the understanding and containment of the current outbreak in non-endemic countries. GeroScience. 2022:1–9. doi: 10.1007/s11357-022-00611-6 35726117 PMC9208705

[12] Damon IK. Status of human monkeypox: clinical disease, epidemiology and research. Vaccine. 2011;29 Suppl 4:D54–9. doi: 10.1016/j.vaccine.2011.04.014 22185831

[13] Jamil H, Tariq W, Tahir MJ, Mahfooz RS, Asghar MS, Ahmed A. Human monkeypox expansion from the endemic to non-endemic regions: Control measures. Annals of medicine and surgery (2012). 2022;79:104048. doi: 10.1016/j.amsu.2022.104048 35860124 PMC9289418

[14] Choudhary G, Prabha PK, Gupta S, Prakash A, Medhi B. Monkeypox infection: A quick glance. Indian journal of pharmacology. 2022;54(3):161–4. doi: 10.4103/ijp.ijp_400_22 35848685 PMC9396685

[15] Hraib M, Jouni S, Albitar MM, Alaidi S, Alshehabi Z. The outbreak of monkeypox 2022: An overview. Annals of medicine and surgery (2012). 2022;79:104069. doi: 10.1016/j.amsu.2022.104069 35860140 PMC9289401

[16] Alshahrani NZ, Alzahrani F, Alarifi AM, Algethami MR, Alhumam MN, Ayied HAM, et al. Assessment of Knowledge of Monkeypox Viral Infection among the General Population in Saudi Arabia. Pathogens (Basel, Switzerland). 2022;11(8). 10.3390/pathogens11080904 PMC9414752 36015025

[17] Mohapatra RK, Tuli HS, Sarangi AK, Chakraborty S, Chandran D, Chakraborty C, et al. Unexpected sudden rise of human monkeypox cases in multiple non-endemic countries amid COVID-19 pandemic and salient counteracting strategies: Another potential global threat? International journal of surgery (London, England). 2022;103:106705. doi: 10.1016/j.ijsu.2022.106705 35697322 PMC9187856

[18] Beer EM, Rao VB. A systematic review of the epidemiology of human monkeypox outbreaks and implications for outbreak strategy. PLoS neglected tropical diseases. 2019;13(10):e0007791. doi: 10.1371/journal.pntd.0007791 31618206 PMC6816577

[19] Ogoina D, Izibewule JH, Ogunleye A, Ederiane E, Anebonam U, Neni A, et al. The 2017 human monkeypox outbreak in Nigeria-Report of outbreak experience and response in the Niger Delta University Teaching Hospital, Bayelsa State, Nigeria. PloS one. 2019;14(4):e0214229. doi: 10.1371/journal.pone.0214229 30995249 PMC6469755

[20] Zumla A, Valdoleiros SR, Haider N, Asogun D, Ntoumi F, Petersen E, et al. Monkeypox outbreaks outside endemic regions: scientific and social priorities. The Lancet Infectious diseases. 2022;22(7):929–31. doi: 10.1016/S1473-3099(22)00354-1 35636447 PMC9141675

[21] Sah R, Mohanty A, Siddiq A, Singh P, Abdelaal A, Alshahrani NZ, et al. Monkeypox reported in India—South East Asia Region: Health and economic challenges. The Lancet regional health Southeast Asia. 2022;4:100063. doi: 10.1016/j.lansea.2022.100063 35991511 PMC9375140

[22] World Health Organization. WHO Director-General declares the ongoing monkeypox outbreak a Public Health Emergency of International Concern 2022, available at: https://www.who.int/europe/news/item/23-07-2022-who-director-general-declares-the-ongoing-monkeypox-outbreak-a-public-health-event-of-international-concern (accessed Sep 15,2022).

[23] World Health Organization. 2022 Monkeypox Outbreak: Global Trends 2022 https://worldhealthorg.shinyapps.io/mpx_global/ (accessed Nov 14, 2022).

[24] World Health Organization. Enhance surveillance, public health measures for monkeypox: WHO 2022, available at: https://www.who.int/southeastasia/news/detail/24-07-2022-enhance-surveillance—public-health-measures-for-monkeypox—who (accessed Sep 18,2022).

[25] REUTERS. Indonesia confirms first monkeypox case in citizen returning from abroad 2022, available at: https://www.reuters.com/world/asia-pacific/indonesia-brief-media-first-confirmed-monkeypox-case-2022-08-20/ (accessed Sep 20,2022).

[26] Abdullah Ali S, Cançado FACQ, de Oliveira CAF. The emergence of monkeypox virus, new challenges to the healthcare settings in Pakistan [published online ahead of print, 2022 Jun 1]. J Med Virol. 2022;e27899. doi: 10.1002/jmv.27899 35642552

[27] No monkeypox case detected in Pakistan yet, clarifies NIH. DAWN news. Report on 24 May 2022. Accessed: Nov 24, 2022. Available from: https://www.dawn.com/news/1691293

[28] World Health Organization. Monkeypox—United Kingdom of Great Britain and Northern Ireland 2022, available at: https://www.who.int/emergencies/disease-outbreak-news/item/2022-DON381 (accessed Sep 20,2022).

[29] Tahir MJ, Saqlain M, Tariq W, Waheed S, Tan SHS, Nasir SI, et al. Population preferences and attitudes towards COVID-19 vaccination: a cross-sectional study from Pakistan. BMC public health. 2021;21(1):1759. doi: 10.1186/s12889-021-11814-5 34565351 PMC8474768

[30] Ahmed A, Saqlain M, Tanveer M, Tahir AH, Ud-Din F, Shinwari MI, et al. Knowledge, attitude and perceptions about Crimean Congo Haemorrhagic Fever (CCHF) among occupationally high-risk healthcare professionals of Pakistan. BMC infectious diseases. 2021;21(1):35. doi: 10.1186/s12879-020-05714-z 33413164 PMC7792042

[31] Bureau of Statics Government of Pakistn. Brief on Census -2017, available at: https://www.pbs.gov.pk/content/brief-census-2017#:~:text=According%20to%20the%20final%20results,males%20and%20101.3%20million%20females (accessed Sep 20,2022).

[32] Riccò M, Ferraro P, Camisa V, Satta E, Zaniboni A, Ranzieri S, et al. When a Neglected Tropical Disease Goes Global: Knowledge, Attitudes and Practices of Italian Physicians towards Monkeypox, Preliminary Results. Tropical medicine and infectious disease. 2022;7(7). doi: 10.3390/tropicalmed7070135 35878146 PMC9316880

[33] Harapan H, Setiawan AM, Yufika A, Anwar S, Wahyuni S, Asrizal FW, et al. Knowledge of human monkeypox viral infection among general practitioners: a cross-sectional study in Indonesia. Pathogens and global health. 2020;114(2):68–75. doi: 10.1080/20477724.2020.1743037 32202967 PMC7170312

[34] World Health Organization. Monkeypox 2022, available at: https://www.who.int/news-room/fact-sheets/detail/monkeypox (accessed Sep 10,2022).

[35] Centers for Disease Control and Prevention. Monkeypox 2022, available at: https://www.cdc.gov/poxvirus/monkeypox/index.html (accessed Aug 10,2022).

[36] Nath SD, Majumder K, Rimti FH, Das J, Tabassum MN, Oishee AN, et al. Assessment of Knowledge on Human Monkeypox Virus among General Population in Bangladesh: A Nationwide Cross-sectional Study. medRxiv. 2022.

[37] Ahmed SK, Abdulqadirb SO, Omar RM, Essa RA, Hussein SH, Khdir AA, et al. Study of knowledge, attitude and anxiety in Kurdistan-region of Iraqi population during the monkeypox outbreak in 2022. 2022.

[38] Durski KN, McCollum AM, Nakazawa Y, Petersen BW, Reynolds MG, Briand S, et al. Emergence of Monkeypox—West and Central Africa, 1970–2017. MMWR Morbidity and mortality weekly report. 2018;67(10):306–10. doi: 10.15585/mmwr.mm6710a5 29543790 PMC5857192

[39] Nolen LD, Osadebe L, Katomba J, Likofata J, Mukadi D, Monroe B, et al. Extended Human-to-Human Transmission during a Monkeypox Outbreak in the Democratic Republic of the Congo. Emerging infectious diseases. 2016;22(6):1014–21. doi: 10.3201/eid2206.150579 27191380 PMC4880088

[40] Hadi AG, Kadhom M, Hairunisa N, Yousif E, Mohammed SA. A review on COVID-19: origin, spread, symptoms, treatment, and prevention. 2020.

[41] Petersen BW, Damon IK. Orthopoxviruses: vaccinia (smallpox vaccine), variola (smallpox), monkeypox, and cowpox. Mandell, Douglas, and Bennett’s principles and practice of infectious diseases 8th ed Philadelphia: Elsevier. 2014.

[42] Kmiec D, Kirchhoff F. Monkeypox: A New Threat? International journal of molecular sciences. 2022;23(14). doi: 10.3390/ijms23147866 35887214 PMC9321130

[43] Bunge EM, Hoet B, Chen L, Lienert F, Weidenthaler H, Baer LR, et al. The changing epidemiology of human monkeypox-A potential threat? A systematic review. PLoS neglected tropical diseases. 2022;16(2):e0010141. doi: 10.1371/journal.pntd.0010141 35148313 PMC8870502

[44] Edghill-Smith Y, Golding H, Manischewitz J, King LR, Scott D, Bray M, et al. Smallpox vaccine-induced antibodies are necessary and sufficient for protection against monkeypox virus. Nature medicine. 2005;11(7):740–7. doi: 10.1038/nm1261 15951823

[45] Earl PL, Americo JL, Wyatt LS, Eller LA, Whitbeck JC, Cohen GH, et al. Immunogenicity of a highly attenuated MVA smallpox vaccine and protection against monkeypox. Nature. 2004;428(6979):182–5. doi: 10.1038/nature02331 15014500

[46] Reynolds MG, Damon IK. Outbreaks of human monkeypox after cessation of smallpox vaccination. Trends in microbiology. 2012;20(2):80–7. doi: 10.1016/j.tim.2011.12.001 22239910

[47] Rimoin AW, Mulembakani PM, Johnston SC, Lloyd Smith JO, Kisalu NK, Kinkela TL, et al. Major increase in human monkeypox incidence 30 years after smallpox vaccination campaigns cease in the Democratic Republic of Congo. Proceedings of the National Academy of Sciences of the United States of America. 2010;107(37):16262–7. doi: 10.1073/pnas.1005769107 20805472 PMC2941342

[48] Malik AA, Winters MS, Omer SB. Attitudes of the US general public towards Monkeypox. bioRxiv. 2022 Jun 21. 10.1371/journal.pone.0278622 PMC9714903 36454991

[49] Askarian M, Danaei M, Vakili V. Knowledge, Attitudes, and Practices Regarding Pandemic H1N1 Influenza Among Medical and Dental Residents and Fellowships in Shiraz, Iran. International journal of preventive medicine. 2013;4(4):396–403. 23671770 PMC3650590

[50] Rehman S, Rantam FA, Batool K, Rahman A, Effendi MH, Rahmahani J, et al. Knowledge, attitude, and practices associated with avian influenza among undergraduate university students of East Java Indonesia: A cross-sectional survey. F1000Research. 2022;11:115. doi: 10.12688/f1000research.74196.1 35340276 PMC8921684

[51] Rudan I. The COVID-19 pandemic: SARS-CoV-2, childhood hepatitis and monkeypox raise five new questions for the global health research community. Journal of global health. 2022;22:01002. doi: 10.7189/jogh.12.01002 35622346 PMC9138816

[52] Ennab F, Nawaz FA, Narain K, Nchasi G, Essar MY. Rise of monkeypox: Lessons from COVID-19 pandemic to mitigate global health crises. Annals of medicine and surgery (2012). 2022;79:104049. doi: 10.1016/j.amsu.2022.104049 35757311 PMC9217059

[53] Shultz JM, Cooper JL, Baingana F, Oquendo MA, Espinel Z, Althouse BM, et al. The Role of Fear-Related Behaviors in the 2013–2016 West Africa Ebola Virus Disease Outbreak. Current psychiatry reports. 2016;18(11):104. doi: 10.1007/s11920-016-0741-y 27739026 PMC5241909

[54] Chan M. Ebola virus disease in West Africa—no early end to the outbreak. The New England journal of medicine. 2014;371(13):1183–5. doi: 10.1056/NEJMp1409859 25140856

[55] Mungmunpuntipantip R, Sriwijitalai W, Wiwanitkit V. Anxiety, depression and monkeypox. Medp Psychiatry Behav Sci. 2022;1(1).

[56] Salim NA, Septadina IS, Permata M. Knowledge, Attitude, and Perception of Anticipating 2022 Global Human Monkeypox Infection among Internal Medicine Residents at Palembang Indonesia: an Online Survey. Jurnal Kedokteran dan Kesehatan: Publikasi Ilmiah Fakultas Kedokteran Universitas Sriwijaya. 2022;9(3):253–62.

[57] Hemati S, Farhadkhani M, Sanami S, Mohammadi-Moghadam F. A review on insights and lessons from COVID-19 to the prevent of monkeypox pandemic. Travel medicine and infectious disease. 2022;50:102441. doi: 10.1016/j.tmaid.2022.102441 36084881 PMC9446553

[58] Bragazzi NL, Kong JD, Mahroum N, Tsigalou C, Khamisy‐Farah R, Converti M, et al. Epidemiological trends and clinical features of the ongoing monkeypox epidemic: A preliminary pooled data analysis and literature review. Journal of Medical Virology. doi: 10.1002/jmv.27931 35692117

[59] Seang S, Burrel S, Todesco E, Leducq V, Monsel G, Le Pluart D, et al. Evidence of human-to-dog transmission of monkeypox virus. Lancet (London, England). 2022;400(10353):658–9. doi: 10.1016/S0140-6736(22)01487-8 35963267 PMC9536767

[60] Girometti N, Byrne R, Bracchi M, Heskin J, McOwan A, Tittle V, et al. Epidemiological Characteristics and Clinical Features of Confirmed Human Monkeypox Virus Cases in Individuals Attending a Sexual Health Centre in London, United Kingdom. 10.1016/S1473-3099(22)00411-X PMC9534773 35785793

[61] Li ZH, Zhang XR, Zhong WF, Song WQ, Wang ZH, Chen Q, et al. Knowledge, attitudes, and practices related to Coronavirus disease 2019 during the outbreak among workers in China: A large cross-sectional study. PLoS neglected tropical diseases. 2020;14(9):e0008584. doi: 10.1371/journal.pntd.0008584 32941447 PMC7498029

[62] Raghupathi V, Raghupathi W. The influence of education on health: an empirical assessment of OECD countries for the period 1995–2015. Archives of public health = Archives belges de sante publique. 2020;78:20. doi: 10.1186/s13690-020-00402-5 32280462 PMC7133023

[63] McArthur L, Peña M, Holbert D. Effects of socioeconomic status on the obesity knowledge of adolescents from six Latin American cities. International journal of obesity and related metabolic disorders: journal of the International Association for the Study of Obesity. 2001;25(8):1262–8. 10.1038/sj.ijo.0801674 11477513

[64] Marmot M. The influence of income on health: views of an epidemiologist. Health affairs (Project Hope). 2002;21(2):31–46. doi: 10.1377/hlthaff.21.2.31 11900185

[65] Deeks A, Lombard C, Michelmore J, Teede H. The effects of gender and age on health related behaviors. BMC public health. 2009;9:213. doi: 10.1186/1471-2458-9-213 19563685 PMC2713232

[66] Mahmood MM, Rehman J, Arif B, Rehman Z, Aasim M, Saeed MT. Knowledge, attitudes and practices of patients with chronic illnesses during the COVID-19 pandemic: A cross-sectional survey from Pakistan. Chronic illness. 2022;18(3):608–19. doi: 10.1177/17423953211023961 34107795 PMC9396733

[67] Ismail NA, Ahmed HA. Knowledge, Attitudes and Practices Related to Avian Influenza among a Rural Community in Egypt. The Journal of the Egyptian Public Health Association. 2010;85(1–2):73–96. 21073849

[68] Junaid Tahir M, Rizwan Siddiqi A, Ullah I, Ahmed A, Dujaili J, Saqlain M. Devastating urban flooding and dengue outbreak during the COVID-19 pandemic in Pakistan. Medical journal of the Islamic Republic of Iran. 2020;34:169. doi: 10.47176/mjiri.34.169 33816368 PMC8004574

[69] Tomar BS, Singh P, Suman S, Raj P, Nathiya D, Tripathi S, et al. Indian community’s knowledge, attitude & practice towards COVID-19. MedRxiv. 2020.

