# Supplementary material for: Socio-demographic determinants of Monkeypox virus preventive behavior: A cross-sectional study in Pakistan
Source: PLoS One. 2023 Aug 10;18(8):e0279952. doi: 10.1371/journal.pone.0279952 (PMC10414588; doi:10.1371/journal.pone.0279952)
Supplement: Attachment — Submitted filename: manuscript comments (1).docx [file pone.0279952.s002.docx]

The manuscript is written well, however, some of the points need to be addressed before its publication. I have mentioned all the points below:

1. Some of the references are not as per the format of the journal. Kindly update the same in the revisions.
2. Read the complete manuscript because some spelling mistakes.
3. There are a few grammatical mistakes in the manuscript, hence I request authors check the manuscript carefully before submitting the revised version of the manuscript

Rest is ok.
