# Supplementary material for: Socio-demographic determinants of Monkeypox virus preventive behavior: A cross-sectional study in Pakistan
Source: PLoS One. 2023 Aug 10;18(8):e0279952. doi: 10.1371/journal.pone.0279952 (PMC10414588; doi:10.1371/journal.pone.0279952)
Supplement: Attachment — Submitted filename: Socio-demographic determinants of monkeypox virus preventive behavior.docx [file pone.0279952.s003.docx]

I have gone through the manuscript, it contains adequate information. However, the English is not satisfactory, serious language editing is required. Sections are well defined but major comments are needed to address to improve this manuscripts.

1. Major language issues found that needs expert attention. Some sentences could be more precise. It seems to be some words that are overused along with article usage issues. Some sentences should be rewritten. The authors should check the punctuation marks because some are missing from the right places.
2. The present title of the article looks proper and concise.
3. The current abstract is concise and focused. Nevertheless, there are also some article (a, an, the) and punctuation usage problems.
4. Please use ***italics*** in the introduction in the genus, species names, and genes.
5. Keyword looks good and adequate.
6. Please follow the journal rules in every major section and subsection; every subsection within major sections will be Level 2 headings. It will be in bold and 16pt font. It is suggested to check the subsections of the Method and result sections. Do not use italic unnecessarily. Only use it in genus and species names, genes, etc. All level 1 heading will be bold and in 18pt font.
7. In the discussion part, Line 266, there is an error in writing. Understand that it could be a typographic problem. So, it ‘that’ should be removed.
8. The tables are clearly labeled, but they need to be ideally positioned. It should be close to the relevant text after the paragraph in which they are first cited.
9. Found some issues in reference citations; please cite references in brackets. For example, “[1], [2-5], or [3, 7, 9].
10. As per journal requirements, Authors should list the supporting information captions at the end of the manuscript in a section titled “Supporting information.” Use Level 1 and bold type for the titles.
11. In line no 303, a letter needs to be included.
