# Supplementary material for: Socio-demographic determinants of Monkeypox virus preventive behavior: A cross-sectional study in Pakistan
Source: PLoS One. 2023 Aug 10;18(8):e0279952. doi: 10.1371/journal.pone.0279952 (PMC10414588; doi:10.1371/journal.pone.0279952)
Supplement: Attachment — Submitted filename: Response to PLOS.docx [file pone.0279952.s004.docx]

4^th^ March 2023

Dear Editor,

*PLOS ONE*

We would like to thank you for considering our manuscript *‘****Socio-demographic determinants of monkeypox virus preventive behavior: a cross-sectional study in Pakistan****’* and for the valuable feedback provided.

We are very grateful for the reviews provided by the editors and each of the external reviewers of this manuscript. The comments are encouraging, and the reviewers appear to share our judgment that this study and its results are important. Please find our detailed response to the comments below in blue. All page numbers refer to the manuscript file with tracked changes. We have also attached the clean files without track changes.

We hope the paper will now be acceptable for publication in the *PLOS ONE.*

Yours Sincerely,

Corresponding Author

**Responses to Reviewer 1**

***Reviewer’s comment***: Some of the references are not as per the format of the journal. Kindly update the same in the revisions.

***Authors’ response****: Dear reviewer, thank you for valuable correction. The changes have been done throughout the manuscript as per your suggestions.*

***Reviewer’s comment***: Read the complete manuscript because some spelling mistakes.

***Authors’ response****: We have revised the manuscript, and corrected spelling mistakes.*

***Reviewer’s comment***: There are a few grammatical mistakes in the manuscript, hence I request authors check the manuscript carefully before submitting the revised version of the manuscript

***Authors’ response****: We have checked the grammatical mistakes and some corrections have been done.*

**Responses to Reviewer 2**

***Reviewer’s comment***: Major language issues found that needs expert attention. Some sentences could be more precise. It seems to be some words that are overused along with article usage issues. Some sentences should be rewritten. The authors should check the punctuation marks because some are missing from the right places.

***Authors’ response***: Dear reviewer, thanks for the comment. We have tried to improve English language.

***Reviewer’s comment***: The present title of the article looks proper and concise.

***Authors’ response****: Dear reviewer, Thank you for your appreciation.*

***Reviewer’s comment***: The current abstract is concise and focused. Nevertheless, there are also some article (a, an, the) and punctuation usage problems.

***Authors’ response****: We have revised the abstract and made some corrections accordingly.*

***Reviewer’s comment***: Please use italics in the introduction in the genus, species names, and genes.

***Authors’ response****: Thanks for correction.* **[Addressed in Page 03; Line No.63-64]**

***Reviewer’s comment***: Keyword looks good and adequate.

***Authors’ response****: Dear reviewer, Thank you for your appreciation.*

***Reviewer’s comment***: Please follow the journal rules in every major section and subsection; every subsection within major sections will be Level 2 headings. It will be in bold and 16pt font. It is suggested to check the subsections of the Method and result sections. Do not use italic unnecessarily. Only use it in genus and species names, genes, etc. All level 1 heading will be bold and in 18pt font.

***Authors’ response****: Correction has been done. Now, we have used bold and 18pt font level 1 headings. Bold and 16pt font level 2 headings.*

***Reviewer’s comment***: In the discussion part, Line 266, there is an error in writing. Understand that it could be a typographic problem. So, it ‘that’ should be removed.

***Authors’ response****: Thanks for correction. The word ‘that’ has been removed now.* **[Addressed in Page 11; Line No.277]**

***Reviewer’s comment***: The tables are clearly labeled, but they need to be ideally positioned. It should be close to the relevant text after the paragraph in which they are first cited.

***Authors’ response****: Thanks for correction. The tables are positioned as per your recommendation.*

***Reviewer’s comment***: Found some issues in reference citations; please cite references in brackets. For example, “[1], [2-5], or [3, 7, 9].

***Authors’ response****: The changes have been done throughout the manuscript as per journal requirements.*

***Reviewer’s comment***: As per journal requirements, Authors should list the supporting information captions at the end of the manuscript in a section titled “Supporting information.” Use Level 1 and bold type for the titles.

***Authors’ response****: The change has been done as per your suggestion.*

***Reviewer’s comment***: In line no 303, a letter needs to be included

***Authors’ response****: The change has been done as per your suggestion.* **[Addressed in Page 19; Line No.373]**
